# Breastfeeding self-efficacy as a mediator between sense of coherence and perception of milk supply among postpartum women: a cross-sectional study

**DOI:** 10.3389/fpubh.2026.1894005

**Published:** 2026-09-18

**Authors:** Duojie Pengmao, Lianhui Wei, Xia'an Sun, Mengyun Hu, Yuhan Zhu, Xuekun Zhang

**Affiliations:** 1 School of Nursing, Suzhou Medical College, Soochow University, Suzhou, Jiangsu, China; 2 Department of Obstetrics, Qinghai Provincial People’s Hospital, Xining, Qinghai, China

**Keywords:** breastfeeding, breastfeeding self-efficacy, H&H Lactation Scale, perceived milk supply, sense of coherence

## Abstract

**Background:**

Concerns about insufficient milk are a common reason for supplementation and breastfeeding cessation. The H&H Lactation Scale (HHLS) captures a broad maternal appraisal of lactation that includes confidence, perceived infant satiety, and satisfaction. We examined the cross-sectional association of sense of coherence with HHLS total and domain scores and estimated the indirect association through breastfeeding self-efficacy.

**Methods:**

This single-center cross-sectional study included 255 women attending a routine one-month postpartum follow-up. Sense of coherence, breastfeeding self-efficacy, and perception of milk supply were measured using the SOC-13, BSES-SF, and Chinese-language HHLS, respectively. Analyses included hierarchical regression, domain-specific path models, and 5,000-resample bootstrap estimates under parsimonious and expanded adjustment specifications. Sensitivity analyses excluded women currently formula feeding.

**Results:**

SOC was positively associated with BSES-SF and HHLS total scores. Adding BSES-SF increased explained variance in HHLS total score from 10.4 to 36.1%. The parsimonious indirect estimate was 0.187 (bootstrap 95% CI 0.097 to 0.299), and the expanded estimate was 0.044 (−0.002 to 0.108). Indirect associations were evident for HHLS Maternal confidence/commitment (0.086, 0.036 to 0.152) and satisfaction (0.102, 0.057 to 0.155), but not infant satiety (−0.001, −0.021 to 0.019). After excluding 48 women currently formula feeding, the corresponding estimates remained positive in both the parsimonious (0.199, 0.102 to 0.323) and expanded (0.061, 0.004 to 0.147) models.

**Conclusion:**

Breastfeeding self-efficacy was related to mothers’ broader appraisal of lactation, particularly confidence and satisfaction, and the principal pattern remained among women who were currently breastfeeding. The findings did not extend to perceived infant satiety and do not establish temporal mediation. BSES-SF may complement, but should not replace, clinical assessment of lactation and infant intake.

## 1 Introduction

Breastfeeding is a central component of maternal and child health policy. The World Health Organization recommends early initiation of breastfeeding, exclusive breastfeeding for the first 6 months of life, and continued breastfeeding with appropriate complementary foods thereafter (1). In recent global health practice, breastfeeding support has increasingly been framed not only as maternal education, but also as an issue of equitable access to skilled counseling, family support, health-system continuity, and protection from barriers that prevent women from sustaining breastfeeding (2). This shift reflects a broader public health consensus that breastfeeding is biologically important but socially conditioned.

The benefits of breastfeeding have been well documented across infant, maternal, and population health outcomes. Breastfeeding is associated with improved infant survival, immune protection, cognitive development, and reduced maternal cardiometabolic risk (3). At the policy level, investment in breastfeeding promotion is considered one of the most cost-effective strategies for improving maternal and child health, but effective implementation requires coordinated action across health services, families, communities, workplaces, and regulatory systems (4). More recent global evidence has also emphasized that breastfeeding practices are increasingly challenged by market forces, social norms, commercial formula promotion, and uneven access to practical lactation support (5). These challenges indicate that breastfeeding continuation cannot be explained by maternal knowledge or motivation alone.

A public health approach to breastfeeding must therefore account for the structural and interpersonal conditions under which women make feeding decisions. The political economy of infant and young child feeding highlights the role of commercial influence, workplace constraints, weak maternity protection, and insufficient public investment in breastfeeding support (6). At the clinical and community levels, breastfeeding outcomes are shaped by timely postpartum counseling, responsive professional assistance, continuity of care, and family-inclusive support (7). Professional policy guidance similarly emphasizes that breastfeeding support should begin before birth, continue through the maternity-care period, and extend into community and primary-care settings after discharge (8). These international practices provide an important background for examining why many women still experience difficulty sustaining exclusive breastfeeding in the early postpartum period.

The challenge is particularly relevant in China. National surveillance has shown progress in breastfeeding practices, but exclusive breastfeeding during the first 6 months remains below recommended public health targets (9). China’s recent child health policy framework also places continued emphasis on improving infant and young child feeding practices as part of broader maternal and child health promotion (10). However, postpartum feeding decisions in China often occur within a multigenerational caregiving context, where mothers, partners, grandparents, and health professionals may all influence infant-feeding choices. In such settings, concerns about infant satiety, weight gain, crying, or frequent feeding can quickly become interpreted as signs that breast milk is insufficient.

Perceived insufficient milk supply is one of the most commonly reported reasons for early breastfeeding cessation and the introduction of formula supplementation (11). A recent systematic review further shows that perceived insufficient milk supply is widespread and shaped by multiple maternal, infant, psychosocial, and health-service factors (12). Earlier evidence from large infant-feeding studies also indicates that mothers frequently report milk-supply concerns when explaining why they stop breastfeeding earlier than intended (13). Similar findings have been reported in studies of earlier-than-desired breastfeeding cessation, where perceived inadequate milk supply remains a dominant reason for stopping breastfeeding (14). These findings suggest that perceived milk supply is not a marginal concern, but a central barrier to sustained breastfeeding.

It is important to distinguish perceived milk insufficiency from objectively insufficient milk production. Mothers may interpret frequent feeding, crying, shorter feeds, softer breasts, cluster feeding, infant sleep changes, or slow but normal growth as signs that milk is inadequate. Theoretical and empirical work has long suggested that maternal confidence, parenting self-efficacy, and interpretation of infant behavior are closely related to perceived milk adequacy (15). Studies among postpartum women also show that breastfeeding self-efficacy is associated with perceived insufficient milk, indicating that confidence may be especially important when mothers evaluate ambiguous feeding cues (16). Evidence from different postpartum populations has further shown that perceived milk supply is closely connected with exclusive breastfeeding practice during the first 6 months (17). Longitudinal research likewise suggests that breastfeeding self-efficacy and perceived milk supply develop together during early postpartum adaptation (18). Clinical studies comparing perceived and actual milk production also indicate that perceived insufficiency may occur even when physiological milk production is not objectively low (19). These findings highlight the need to examine the psychosocial basis of perceived milk supply.

Sense of coherence provides a useful theoretical lens for understanding maternal adaptation during the postpartum period. Antonovsky first developed the salutogenic model to explain how individuals manage stress and maintain health under challenging conditions (20). He later conceptualized sense of coherence as a global orientation through which people perceive life events as comprehensible, manageable, and meaningful (21). The SOC scale has been widely validated across populations and health contexts (22), and systematic evidence suggests that stronger sense of coherence is associated with better health and adaptive functioning (23). Health-promotion scholarship has further emphasized that sense of coherence is not merely a descriptive psychological construct, but a framework for understanding how people mobilize resources and make sense of stressful experiences (24). In early motherhood, this perspective is relevant because lactation establishment involves bodily change, emotional demands, uncertain infant signals, and advice from multiple sources.

Existing research suggests that sense of coherence may be relevant to breastfeeding and perinatal experience. A recent scoping review has identified sense of coherence as a potentially important construct among breastfeeding women, while also noting the need for more focused empirical studies (25). Research on early weaning has reported an association between women’s sense of coherence and breastfeeding continuation (26). Qualitative and mixed-methods evidence has also linked women’s sense of coherence to how they interpret and manage infant-feeding experiences (27). In the broader perinatal context, sense of coherence has been associated with maternal fear, coping, and adjustment during the transition to motherhood (28). However, the specific relationship between sense of coherence and perceived milk adequacy remains insufficiently examined.

Sense of coherence alone is unlikely to account directly for how mothers evaluate milk adequacy because it is a broad orientation toward managing life demands rather than a feeding-specific judgment. Breastfeeding self-efficacy offers a theoretically closer construct. Bandura’s self-efficacy theory proposes that confidence in performing a behavior is related to effort, persistence, emotional response, and recovery after setbacks (29). His later work established efficacy beliefs as central to behavioral regulation and human agency (30). The Breastfeeding Self-Efficacy Scale was developed to assess mothers’ perceived capability to breastfeed successfully (31), and its short form captures confidence in technical and intrapersonal aspects of breastfeeding (32). Multinational psychometric evidence further supports the BSES-SF across diverse groups of new mothers (33). In the present framework, sense of coherence represents a broad coping resource, whereas breastfeeding self-efficacy represents the domain-specific appraisal most immediately aligned with interpreting feeding difficulty and milk adequacy.

Evidence from intervention research indicates that breastfeeding self-efficacy is potentially responsive to education, skills practice, feedback, modeling, and continued support (34). Theory-based educational interventions have also been associated with stronger breastfeeding self-efficacy and higher exclusive breastfeeding rates (35). More broadly, skilled counseling and continuing support can improve breastfeeding outcomes across clinical and community settings (36), while structured support is beneficial for healthy breastfeeding mothers with healthy term infants (37). Digital and mobile health approaches have also been investigated as ways to provide timely information and address perceived insufficient milk (38). These studies establish the practical relevance of breastfeeding self-efficacy, but they do not by themselves determine whether self-efficacy temporally precedes perceived milk supply in a cross-sectional sample.

The H&H Lactation Scale was developed to assess mothers’ appraisal of lactation across breastfeeding confidence and determination, perceived infant satiety, and Maternal-infant breastfeeding (39). Subsequent research has used the scale to examine maternal perceptions of milk adequacy in clinical settings (40). This multidimensional structure is informative, but it also requires careful terminology. The HHLS total score represents a broad perception of milk supply rather than a narrow estimate of milk production, and its Maternal confidence/commitment domain is conceptually close to breastfeeding self-efficacy. Analyses based only on the total score may therefore combine distinct perceptions of infant satiety with confidence and satisfaction.

Breastfeeding self-efficacy develops within a wider psychosocial and caregiving context. Postnatal depressive symptoms may be associated with how mothers interpret infant cues, evaluate competence, and respond to breastfeeding difficulty (41). Infant-feeding attitudes may be related to persistence and responses to uncertainty (42), while perceived social support can provide reassurance, practical assistance, and access to problem-solving resources (43). Recent evidence from Italian mothers further indicates that breastfeeding self-efficacy is jointly associated with feeding practices, general maternal self-efficacy, postpartum information, parity, parental stress, and, less consistently, attachment (44). This integrated evidence suggests that breastfeeding confidence should not be treated as an isolated individual trait. It also clarifies why social support was examined as a contextual correlate and sensitivity variable rather than specified as the primary mediator: support can shape confidence and feeding experience, whereas BSES-SF directly measures mothers’ perceived capability to breastfeed.

Despite growing evidence on breastfeeding confidence and perceived milk adequacy, three questions remain. First, broad coping orientation and breastfeeding-specific confidence have rarely been examined within the same empirical framework. Second, the conceptual distinction between breastfeeding self-efficacy and multidimensional perception of milk supply requires direct examination because the HHLS includes confidence-related content. Third, cross-sectional studies must allow for reciprocal relationships among feeding experience, confidence, intentions, mood, and support. A defensible analysis should therefore report both the HHLS total score and its domains, distinguish theory-informed ordering from temporal mediation, and examine how estimates change under alternative covariate sets.

This study examined the cross-sectional associations among sense of coherence, breastfeeding self-efficacy, and perception of milk supply among postpartum women. We expected stronger sense of coherence to be associated with higher breastfeeding self-efficacy and more favorable HHLS scores. We also examined the HHLS domains separately, with particular attention to perceived infant satiety because it has less direct conceptual overlap with BSES-SF. Finally, we assessed the sensitivity of the indirect association to alternative adjustment specifications. The analyses do not establish temporal or causal ordering.

## 2 Materials and methods

### 2.1 Study design and analytic framework

This single-center cross-sectional study examined a theory-informed statistical ordering among sense of coherence, breastfeeding self-efficacy, and perception of milk supply. Sense of coherence was treated as a broad salutogenic orientation, BSES-SF as a breastfeeding-specific confidence measure, and HHLS as a multidimensional maternal appraisal containing Maternal confidence/commitment, infant satiety, and satisfaction. Because BSES-SF and one HHLS domain assess related confidence content, the total-score model was accompanied by domain-level analyses rather than being treated as evidence that the constructs were fully distinct. Reporting followed STROBE recommendations (45).

The primary analysis estimated the cross-sectional indirect association between SOC and HHLS total score through BSES-SF. Figure 1 presents the theory-informed ordering and the covariate framework. The parsimonious model included education, parity, and expected breastfeeding duration; the expanded model added background, obstetric, feeding, and concurrent psychosocial variables. Neither specification was regarded as uniquely correct. Expected breastfeeding duration was measured concurrently with the study scales and may itself reflect current feeding experience, confidence, and perceived adequacy.

**Figure 1:**
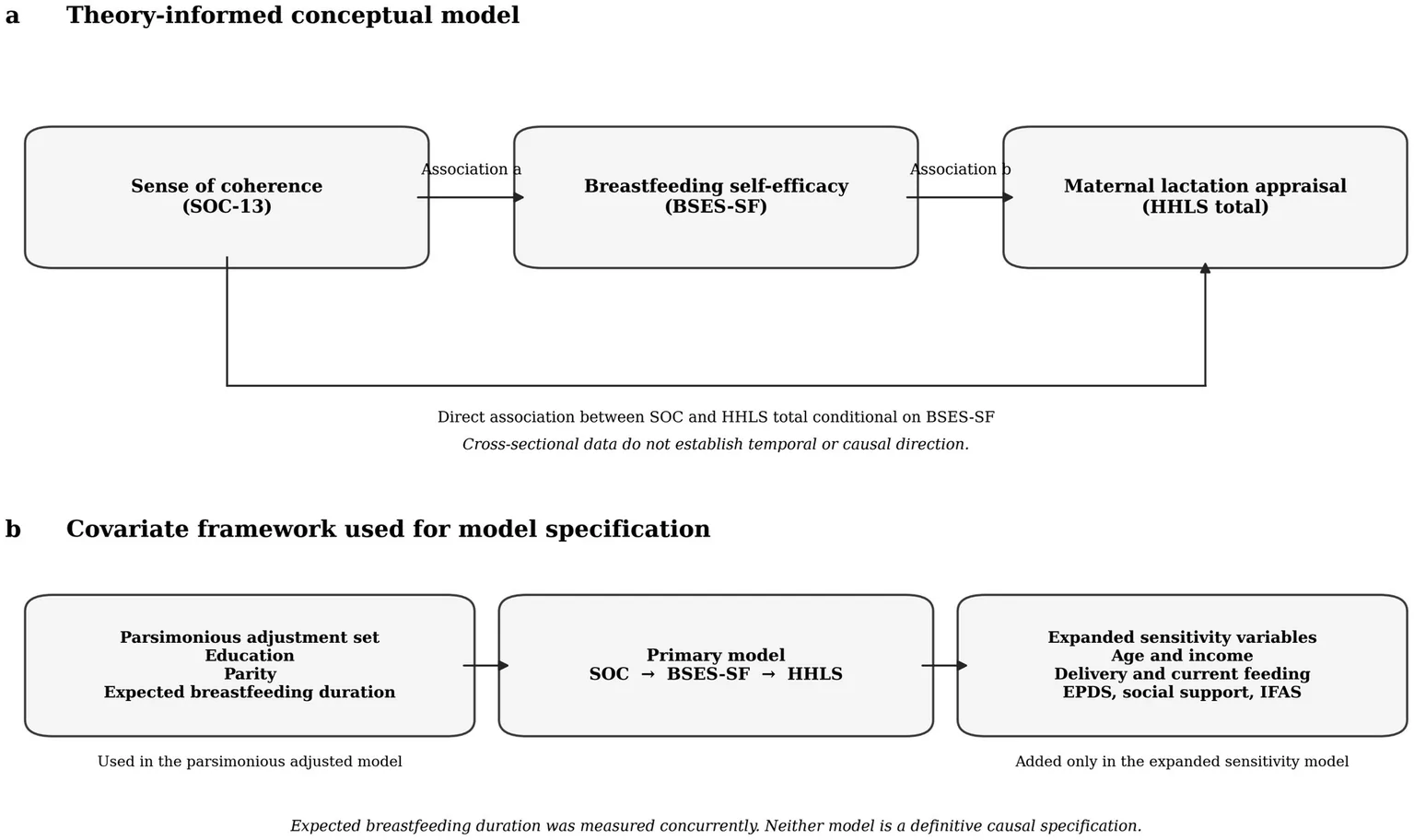
Theory-informed cross-sectional model and covariate framework. Panel **(a)** presents the statistical ordering among SOC, BSES-SF, and HHLS. Panel **(b)** distinguishes the parsimonious adjustment set from variables added in the expanded sensitivity model. Expected breastfeeding duration was measured concurrently, and the figure does not assert temporal or causal direction.

### 2.2 Sample size considerations

The recruitment target was based on the number of eligible postpartum women available during the study period; no formal *a priori* power calculation was undertaken. A sensitivity calculation for the final sample indicated that 255 observations provided approximately 80% power, at a two-sided alpha of 0.05, to detect a Cohen’s f^2^ of approximately 0.071 in a multiple regression model with 12 predictors. This calculation supports the ability to detect small-to-moderate overall regression effects, but it does not guarantee adequate precision for subgroup or dimension-specific indirect estimates. Those analyses were therefore treated as exploratory.

### 2.3 Participants and setting

Participants were recruited from April to July 2025 at the routine postpartum follow-up service of Qinghai Provincial People’s Hospital in Xining, Qinghai Province, China. The hospital serves women from urban Xining and geographically dispersed, ethnically diverse communities. Eligibility required a singleton term birth, gestational age of at least 37 weeks, infant birth weight of at least 2,500 g, ability to complete the questionnaire, and written informed consent. The assessment was conducted at the scheduled one-month postpartum follow-up; the exact postpartum day was not retained in the analytic dataset. Recruitment from one tertiary hospital was not intended to produce a province-representative sample.

Women were excluded for severe maternal disease, infectious disease, a documented psychiatric disorder, special infant-feeding requirements, mother-infant separation, or explicit refusal to breastfeed that prevented meaningful assessment of the study constructs. During the April–July 2025 recruitment period, 280 questionnaires were distributed and 255 complete questionnaires met the prespecified completeness and consistency criteria and were included in analysis, yielding an analytic response of 91.1%. The study log did not distinguish non-return from incomplete or inconsistent questionnaires among the 25 not included; these records are therefore reported collectively rather than assigned unsupported individual reasons. The available participant flow is shown in Supplementary Figure S1.

### 2.4 Questionnaire administration and quality control

All questionnaires were administered in simplified Chinese. Participants completed the questionnaires independently, with trained research staff available to explain procedural requirements or clarify literal wording without suggesting answers. Validated Chinese-language versions were used; no additional translation or back-translation was undertaken for this study. No minority-language versions were administered, and eligibility required sufficient Chinese communication and reading ability to complete the questionnaire.

Returned questionnaires were reviewed for missing pages, implausible response patterns, and inconsistent demographic information. Records were included only when all primary scale scores and model covariates could be calculated. The final analytic sample contained no missing values for prespecified variables. Because the original study log did not retain item-level missingness for the 25 questionnaires not included, no further subdivision of those records was possible. Standardized instructions, privacy, and separation of questionnaire completion from clinical decision-making were used to reduce response contamination, although common method variance remains possible.

### 2.5 Measures and scale scoring

#### 2.5.1 Sense of coherence

Sense of coherence was the primary exposure and was assessed with the 13-item Sense of Coherence Scale (SOC-13). The instrument represents comprehensibility, manageability, and meaningfulness, and a Chinese version has demonstrated acceptable psychometric performance (46). Comprehensibility concerns whether internal and external stimuli are perceived as structured and understandable; manageability reflects perceived access to coping resources; and meaningfulness concerns whether demands are regarded as worthy of engagement. The construct and its scoring are consistent with the salutogenic measurement framework (47). Items were coded and summed to produce total and dimension scores, with higher scores indicating stronger sense of coherence. The total score was prespecified for primary analyses because it provides the most stable representation of the construct.

#### 2.5.2 Breastfeeding self-efficacy

Breastfeeding self-efficacy was the intermediary variable specified in the path model and was assessed with the 14-item Breastfeeding Self-Efficacy Scale-Short Form (BSES-SF). The scale assesses confidence in breastfeeding-related technical and intrapersonal capacities and has shown prognostic and psychometric utility among Mandarin-speaking Chinese mothers (48). Its selection was also grounded in self-efficacy theory, which links perceived capability with effort, persistence, and responses to difficulty (49). Items were summed to produce technical, intrapersonal, and total scores, with higher values indicating stronger breastfeeding self-efficacy. The total score was used for primary analyses.

#### 2.5.3 Perception of milk supply

Perception of milk supply was assessed using the 20-item Chinese-language H&H Lactation Scale (HHLS). The instrument comprises maternal confidence/commitment, perceived infant satiety, and maternal-infant breastfeeding satisfaction, with higher scores indicating a more favorable perception of milk supply (50). The Chinese version used in mainland postpartum research retained the 20-item, three-domain structure and showed a content validity index of 0.98 and Cronbach’s alpha of 0.93 (51). In the present sample, Cronbach’s alpha was 0.875 for the total score and 0.850, 0.741, and 0.919 for the three domains. Because Maternal confidence/commitment overlaps conceptually with BSES-SF, the total-score analysis was accompanied by domain-specific path models, with infant satiety treated as the least overlapping outcome.

#### 2.5.4 Additional psychosocial measures

Postnatal depressive symptoms were assessed with the validated Mainland Chinese EPDS; scores greater than 10 were classified as elevated depression risk for descriptive analyses (52). Breastfeeding attitude was assessed with the Mainland Chinese Iowa Infant Feeding Attitude Scale (53), and Breastfeeding social support was assessed using the Breastfeeding Social Support Scale (BSSS) (54). These variables were treated as concurrent psychosocial measures rather than clearly antecedent covariates.

### 2.6 Covariates

Covariates were organized by temporal and substantive role. Selection was informed by a review of breastfeeding self-efficacy measures (55) and systematic evidence on breastfeeding determinants (56). Background characteristics included age, ethnicity, education, pre-pregnancy body mass index, household income, employment status, residence, and parity. Ethnicity was considered because feeding norms and access may differ across population groups (57). Education may reflect health literacy and access to breastfeeding information (58), employment and income may capture work and material constraints (59), and pre-pregnancy BMI may be related to lactation initiation and duration (60). Education and parity were retained in the parsimonious model to preserve the prespecified analysis. A separate sensitivity model adjusted for age, ethnicity, education, pre-pregnancy BMI category, employment, residence, parity, and delivery mode.

Obstetric and current feeding characteristics included parity, mode of delivery, feeding mode at the survey, and expected breastfeeding duration. Parity may capture prior feeding experience (61). Expected duration was measured concurrently and may reflect both intention and current experience (62). Cesarean delivery may affect early lactation establishment (63), while current feeding and Maternal-infant breastfeeding are closely interrelated with self-efficacy (64). Feeding mode was retained as three distinct descriptive categories: exclusive breastfeeding, mixed feeding, and formula feeding. The parsimonious model included education, parity, and expected duration to match the prespecified analysis, while interpretation recognizes that expected duration may be a consequence or correlate of current feeding experience.

Concurrent psychosocial variables included EPDS, IFAS, and perceived social support. Depressive symptoms may develop reciprocally with breastfeeding experience (65), feeding attitudes may reflect both intention and experience (66), and social support is closely related to breastfeeding self-efficacy (67). The expanded model additionally included age, income, delivery mode, current exclusive breastfeeding, EPDS, IFAS, and social support. These variables could represent confounders, concurrent correlates, alternative intermediaries, downstream variables, or, under some causal structures, colliders. The expanded model was therefore used to show specification sensitivity rather than to identify a definitive causal adjustment set (68, 69).

### 2.7 Scale scoring and data management

Scale items were oriented so that higher values indicated stronger sense of coherence, greater breastfeeding self-efficacy, more favorable perception of milk supply, stronger social support, more favorable breastfeeding attitudes, or more depressive symptoms, as appropriate. Reverse-coded items were recoded before totals and domains were calculated. Scale totals and domain scores were analyzed continuously, consistent with established treatment of multi-item Likert measures (70). SOC categories of 13–63, 64–79, and 80–91 were used only for descriptive presentation. The dichotomous perceived-milk-status variable was removed because its source, coding, and validation could not be verified.

The analytic dataset was checked for completeness, plausible ranges, and consistency between item-level and scale-level values. All 255 retained records had calculable SOC-13, BSES-SF, HHLS, and planned covariate values, so complete-case analysis was used with attention to its selection assumptions (71, 72). Formula feeding described current feeding mode at assessment. The questionnaire did not preserve whether these women had initiated breastfeeding, the timing or reason for discontinuation, or a separate “not applicable” response option. To assess the influence of this uncertainty, the parsimonious and expanded path models were repeated after excluding the 48 women currently formula feeding.

### 2.8 Statistical analysis

Continuous variables were summarized using means and standard deviations and, where informative, medians and interquartile ranges. Categorical variables were summarized using counts and percentages. Internal consistency was evaluated using Cronbach’s alpha. Pearson correlations characterized bivariate associations. Welch’s *t*-tests and one-way analysis of variance were used for descriptive group comparisons. SOC categories and the EPDS risk category were used only in these descriptive comparisons.

Hierarchical linear regression quantified incremental variance in HHLS total scores. Model 1 included SOC; Model 2 added BSES-SF; Model 3 added education, parity, and expected breastfeeding duration; and Model 4 added age, ordinal household income, delivery mode, current exclusive breastfeeding, EPDS, social support, and IFAS. *R*^2^, adjusted *R*^2^, and change in *R*^2^ were reported. Age was entered continuously; education, parity, delivery mode, and exclusive breastfeeding were binary; household income and expected breastfeeding duration were entered as ordered scores. The equal-spacing assumption for ordinal predictors is a simplifying model assumption and is documented in Supplementary Table S4.

Ordinary least squares path models estimated path a from SOC to BSES-SF, path b from BSES-SF to HHLS conditional on SOC, the total association c, the direct association c′, and the product a × b. Percentile bootstrap confidence intervals used 5,000 resamples with a fixed random seed (20260728) and are reported to three decimal places. Crude, parsimonious adjusted, and expanded sensitivity models were estimated. The same parsimonious path model was repeated for each HHLS domain and among women currently reporting exclusive or mixed feeding. A background-characteristic sensitivity model additionally adjusted for age, ethnicity, education, BMI category, employment, residence, parity, and delivery mode.

Model checks included residual-versus-fitted and Q−Q plots, the Jarque−Bera test, the Breusch−Pagan test, leverage, Cook’s distance, variance inflation factors, HC3 heteroscedasticity-robust standard errors, and a sensitivity refit excluding observations above the heuristic Cook’s distance threshold of 4/n. Exploratory subgroup models were removed because no formal interaction tests had been prespecified.

### 2.9 Ethics

The study involved human participants. The study protocol and study documents were approved prospectively by the Research Ethics Committee of Qinghai Provincial People’s Hospital under Research Ethics Review No. 科研伦审(2025)-247–02 on 27 March 2025, before recruitment began in April 2025. This approved protocol governed participant recruitment and questionnaire collection from April to July 2025. All participants provided written informed consent before questionnaire completion, and the analytic dataset was de-identified before analysis.

## 3 Results

### 3.1 Participant characteristics and feeding profile

The analytic sample comprised 255 postpartum women recruited from April to July 2025. Most were aged 20–34 years (86.3%); 60.8% identified as Han and 39.2% as an ethnic minority; and 86.7% had college education or above. Most had a normal pre-pregnancy BMI (65.9%), lived in urban areas (80.4%), and reported fixed employment (66.7%). The exact postpartum day was not recorded, so assessment timing could not be compared across feeding groups or added to the regression models.

Two-thirds of participants were primiparous, 58.4% had a vaginal birth, and 41.6% had a cesarean birth. At the survey, 138 women (54.1%) reported exclusive breastfeeding, 69 (27.1%) mixed feeding, and 48 (18.8%) formula feeding. Formula-feeding participants were not merged with mixed-feeding participants in descriptive reporting. The study record did not distinguish women who had never established breastfeeding from those who had discontinued after initiation. Full characteristics are shown in Table 1.

**Table 1:** Sociodemographic, obstetric, and feeding characteristics of participants.

Variable	Category	*n*	%
Age category	<20 years	1	0.4
	20–34 years	220	86.3
	≥35 years	34	13.3
Ethnicity	Han	155	60.8
	Ethnic minority	100	39.2
Education	High school or below	34	13.3
	College or above	221	86.7
Pre-pregnancy BMI	Underweight	40	15.7
	Normal	168	65.9
	Overweight	40	15.7
	Obesity	7	2.7
Monthly household income	≤5,000 CNY	64	25.1
	5,000–10,000 CNY	126	49.4
	≥10,000 CNY	65	25.5
Employment status	Unemployed	24	9.4
	Flexible work	61	23.9
	Fixed employment	170	66.7
Residence	Rural	50	19.6
	Urban	205	80.4
Parity	Primiparous	170	66.7
	Multiparous	85	33.3
Mode of delivery	Vaginal birth	149	58.4
	Cesarean birth	106	41.6
Feeding mode at survey	Formula feeding	48	18.8
	Mixed feeding	69	27.1
	Exclusive breastfeeding	138	54.1
Expected breastfeeding duration	<6 months	88	34.5
	6–12 months	154	60.4
	1–2 years	12	4.7
	≥2 years	1	0.4
Depression risk	No risk	162	63.5
	At risk	93	36.5

BMI, body mass index; CNY, Chinese yuan. Depression risk was defined as EPDS >10. Feeding mode refers to current feeding at the survey. Percentages may not sum to 100 because of rounding.

### 3.2 Scale descriptives and internal consistency

The primary psychosocial scales showed adequate variability for subsequent correlation, regression, and path analyses. The mean SOC total score was 62.88 (SD 12.60), with a median of 64.00 (IQR 54.00–72.00). The mean BSES-SF total score was 50.78 (SD 12.94), with a median of 53.00 (IQR 42.00–58.50). The mean HHLS total score was 104.79 (SD 17.23), with a median of 105.00 (IQR 95.50–117.00). The distributions of SOC, BSES-SF, and HHLS total scores showed sufficient spread and no severe floor or ceiling effect (Figure 2). This pattern indicated that the three primary constructs could be treated as continuous variables in the planned analyses.

**Figure 2:**
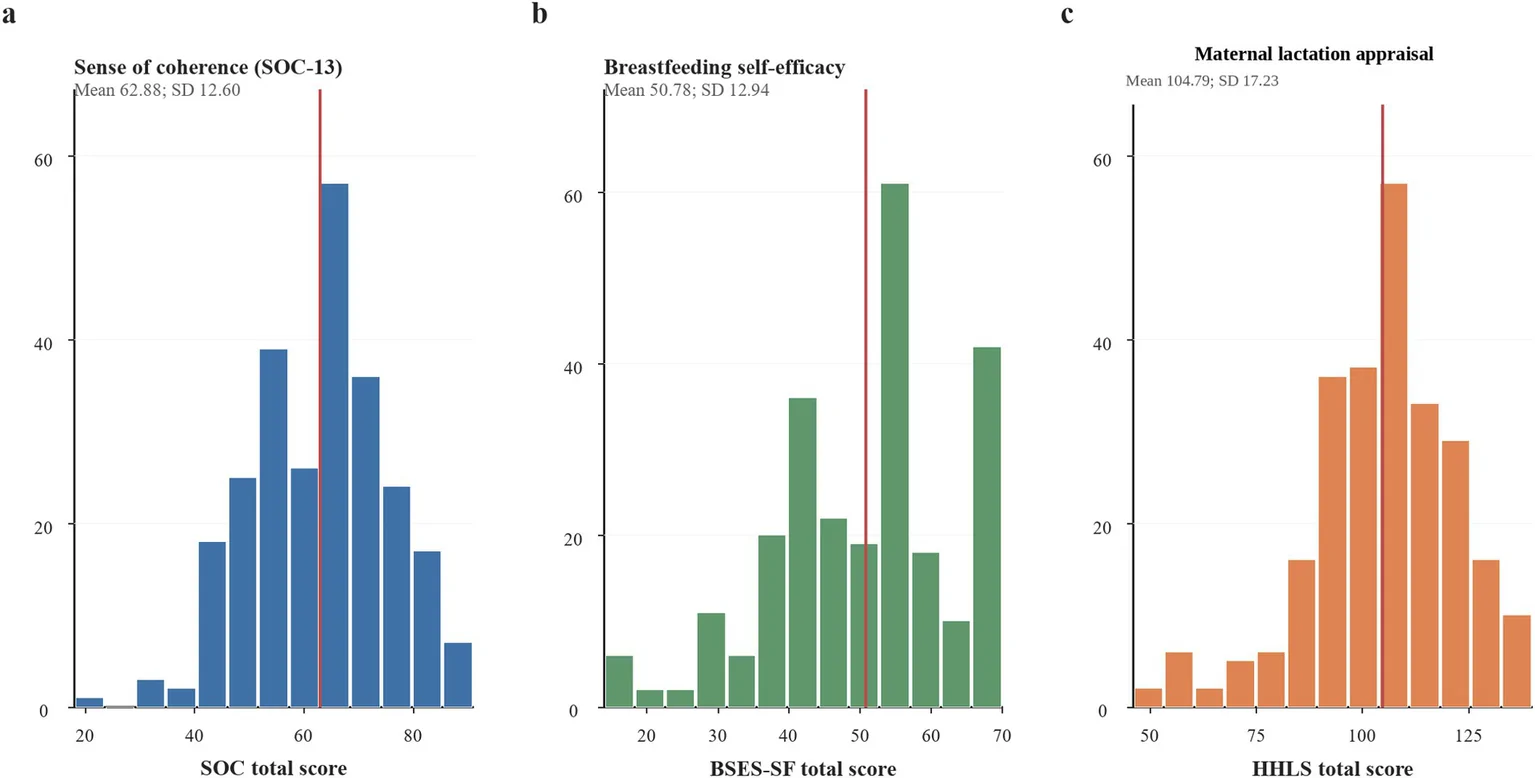
Distributions of **(a)** SOC-13 total scores, **(b)** BSES-SF total scores, and **(c)** HHLS total scores. Red vertical lines indicate the mean.

Internal consistency was acceptable to excellent for the primary total scores. Cronbach’s alpha was 0.876 for SOC, 0.974 for BSES-SF, and 0.875 for HHLS. The BSES-SF technical and intrapersonal dimensions had alpha values of 0.963 and 0.950, and HHLS subscale values ranged from 0.741 to 0.919. SOC comprehensibility and manageability had alpha values of 0.704 and 0.734, whereas meaningfulness had lower internal consistency (alpha = 0.592). Accordingly, the SOC total score was used for primary inference, and meaningfulness-specific estimates were treated as hypothesis-generating rather than confirmatory. Full descriptives are presented in Table 2.

**Table 2:** Scale descriptives and internal consistency.

Instrument	Subscale	Range	Mean (SD)	Median (IQR)	Cronbach alpha
SOC-13	Comprehensibility	5–35	23.38 (5.16)	23.00 (19.00–27.50)	0.704
SOC-13	Manageability	4–28	19.04 (4.76)	20.00 (15.00–23.00)	0.734
SOC-13	Meaningfulness	4–28	20.46 (3.92)	20.00 (18.00–23.00)	0.592
SOC-13	Total score	13–91	62.88 (12.60)	64.00 (54.00–72.00)	0.876
BSES-SF	Technical dimension	7–35	25.55 (6.85)	27.00 (21.00–30.00)	0.963
BSES-SF	Intrapersonal dimension	7–35	25.23 (6.47)	27.00 (21.00–28.50)	0.950
BSES-SF	Total score	14–70	50.78 (12.94)	53.00 (42.00–58.50)	0.974
HHLS	Breastfeeding Maternal confidence/commitment	10–70	56.14 (10.11)	58.00 (52.00–64.00)	0.850
HHLS	Infant satiety perception	5–35	21.14 (6.20)	20.00 (17.00–26.00)	0.741
HHLS	Maternal-infant breastfeeding	5–35	27.51 (6.31)	28.00 (24.00–32.00)	0.919
HHLS	Total score	20–140	104.79 (17.23)	105.00 (95.50–117.00)	0.875
IFAS	Total score	17–85	65.60 (11.15)	66.00 (60.00–75.00)	0.894
BSSS	Total score	11–55	46.67 (9.16)	48.00 (44.00–55.00)	0.965
EPDS	Total score	0–30	8.58 (6.10)	8.00 (5.00–11.00)	0.905

SOC-13, 13-item Sense of Coherence Scale; BSES-SF, Breastfeeding Self-Efficacy Scale-Short Form; HHLS, H&H Lactation Scale; IFAS, Iowa Infant Feeding Attitude Scale; EPDS, Edinburgh Postnatal Depression Scale; IQR, interquartile range; BSSS, Breastfeeding Social Support Scale.

### 3.3 Psychosocial correlations with perception of milk supply

The SOC was positively correlated with BSES-SF (*r* = 0.28, *p* < 0.001) and HHLS total score (*r* = 0.32, *p* < 0.001). BSES-SF had a stronger correlation with HHLS total score (*r* = 0.58, *p* < 0.001), but this coefficient should be interpreted in light of shared confidence-related content. Social support was positively correlated with SOC (*r* = 0.35), BSES-SF (*r* = 0.60), and HHLS total score (*r* = 0.54), all *p* < 0.001.

Depressive symptoms were weakly negatively correlated with SOC (*r* = −0.16, *p* < 0.01) and were not clearly correlated with HHLS total score (*r* = −0.07). IFAS was weakly correlated with HHLS total score (*r* = 0.16, *p* < 0.05). The complete matrix is reported in Table 3 and Figure 3; domain-level associations are presented in Section 3.7 and Table 4.

**Table 3:** Pearson correlations among SOC, BSES-SF, HHLS total, IFAS, perceived social support, and EPDS.

Variable	SOC	BSES	HHLS	IFAS	Social support	EPDS
SOC	1					
BSES	0.28***	1				
HHLS	0.32***	0.58***	1			
IFAS	0.03	0.08	0.16*	1		
Social support	0.35***	0.60***	0.54***	0.07	1	
EPDS	−0.16**	0.07	−0.07	−0.13*	0.06	1

Values are Pearson correlation coefficients. *p < 0.05, **p < 0.01, ***p < 0.001. SOC, sense of coherence; BSES-SF, Breastfeeding Self-Efficacy Scale-Short Form; HHLS, H&H Lactation Scale; IFAS, Iowa Infant Feeding Attitude Scale; EPDS, Edinburgh Postnatal Depression Scale.

**Figure 3:**
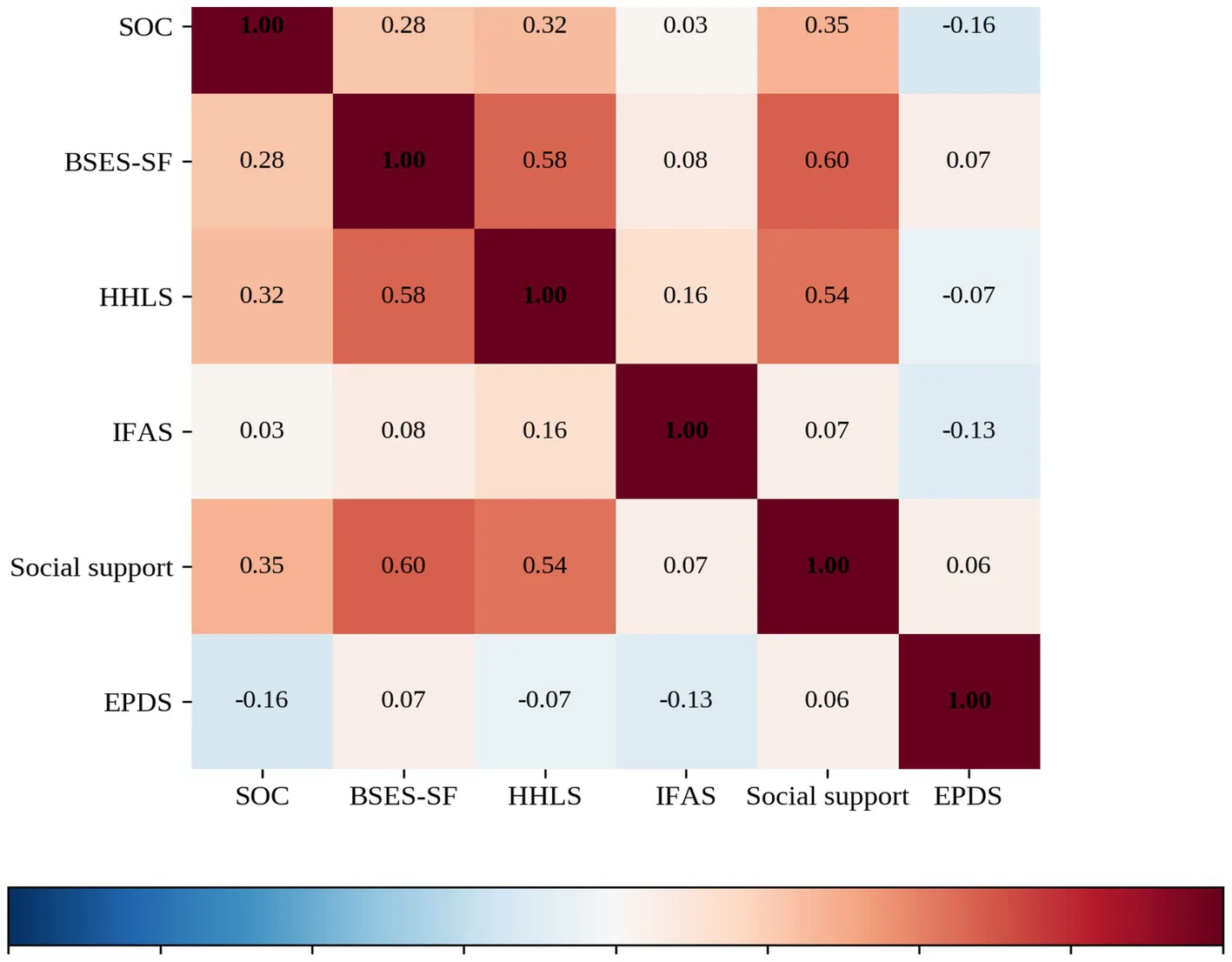
Correlation structure of SOC, BSES-SF, HHLS, IFAS, perceived social support, and EPDS. Cells show Pearson correlation coefficients.

**Table 4:** Domain-specific cross-sectional indirect associations through BSES-SF.

Outcome	Path b (BSES-SF → outcome)	Indirect association	Bootstrap 95% CI	Interpretive focus
HHLS total	0.661	0.187	0.097 to 0.299	Overall perception of milk supply
Maternal confidence/commitment	0.304	0.086	0.036 to 0.152	Confidence-related appraisal
Perceived infant satiety	−0.004	−0.001	−0.021 to 0.019	Infant-cue appraisal
Maternal-infant breastfeeding satisfaction	0.361	0.102	0.057 to 0.155	Satisfaction with lactation

Models adjusted for education, parity, and expected breastfeeding duration. Estimates are unstandardized and bootstrap confidence intervals are based on 5,000 resamples.

### 3.4 Descriptive group differences in perception of milk supply

The HHLS total scores differed across selected education, parity, expected breastfeeding duration, SOC, and depression-risk groups (Table 5). Women with high school education or below had higher unadjusted scores than those with college education or above, but the lower-education subgroup was small. Multiparous women had higher scores than primiparous women, and HHLS scores increased across the descriptive SOC categories.

**Table 5:** Descriptive group comparisons of perception of milk supply measured by HHLS total score.

Variable	Category	*n*	HHLS mean (SD)	Statistic	*p*
Education	High school or below	34	110.09 (15.67)	*t* = 2.09	0.043
	College or above	221	103.98 (17.34)		
Parity	Primiparous	170	102.86 (16.96)	*t* = −2.55	0.012
	Multiparous	85	108.66 (17.21)		
Expected breastfeeding duration	<6 months	88	100.66 (16.63)	*F* = 4.08	0.018
	6–12 months	154	106.79 (16.90)		
	≥1 year	13	109.15 (20.92)		
SOC level	Low	176	101.66 (17.35)	*F* = 11.75	<0.001
	Moderate	55	109.60 (14.87)		
	High	24	116.75 (13.77)		
Depression risk	No risk	162	106.83 (17.61)	*t* = 2.59	0.010
	At risk	93	101.24 (16.02)		
Mode of delivery	Vaginal birth	149	105.05 (17.17)	*t* = 0.29	0.775
	Cesarean birth	106	104.42 (17.39)		

Two-group comparisons used Welch’s *t*-test; comparisons with three or more groups used one-way analysis of variance. SOC categories were used only for descriptive presentation. Depression risk was defined as EPDS >10. The original expected-duration categories of 1–2 years and ≥2 years were combined because the latter contained one participant.

Women with EPDS scores of 10 or lower reported higher HHLS total scores than women with EPDS scores above 10. Delivery mode was not associated with HHLS total score. Feeding-mode comparisons were not included in Table 5 because current feeding may be both a determinant and a consequence of perception of milk supply, and because the source data did not retain the breastfeeding histories needed to interpret formula feeding. All comparisons were unadjusted and descriptive.

### 3.5 Hierarchical models of perception of milk supply

Hierarchical regression showed progressively greater explained variance in HHLS total score (Table 6). Model 1 explained 10.4% of the variance. Adding BSES-SF in Model 2 increased *R*^2^ to 36.1% (Δ*R*^2^ = 0.257). Adding education, parity, and expected breastfeeding duration in Model 3 increased R^2^ by 0.017, and the expanded Model 4 increased it by a further 0.075 to 45.3%. These increments quantify model fit rather than causal contribution.

**Table 6:** Hierarchical linear regression models predicting HHLS total perception of milk supply.

Model	Predictor	*B* (SE)	95% CI	*p*	*R*^2^ (Δ*R*^2^)	Adjusted *R*^2^
Model 1	SOC total	0.44 (0.08)	0.28 to 0.60	<0.001	0.104	0.100
Model 2	SOC total	0.24 (0.07)	0.10 to 0.38	0.001	0.361 (0.257)	0.356
	BSES-SF total	0.70 (0.07)	0.57 to 0.84	<0.001		
Model 3	SOC total	0.24 (0.07)	0.10 to 0.38	<0.001	0.378 (0.017)	0.365
	BSES-SF total	0.66 (0.07)	0.52 to 0.80	<0.001		
	College or above	−3.65 (2.57)	−8.68 to 1.39	0.157		
	Multiparous	2.21 (1.87)	−1.46 to 5.88	0.239		
	Expected BF duration	2.58 (1.54)	−0.43 to 5.60	0.094		
Model 4	SOC total	0.15 (0.07)	0.01 to 0.29	0.040	0.453 (0.075)	0.425
	BSES-SF total	0.45 (0.08)	0.29 to 0.61	<0.001		
	College or above	−4.31 (2.60)	−9.40 to 0.79	0.099		
	Multiparous	3.84 (1.90)	0.13 to 7.56	0.044		
	Expected BF duration	2.35 (1.46)	−0.52 to 5.23	0.109		
	Age	−0.18 (0.22)	−0.62 to 0.25	0.408		
	Monthly income	0.99 (1.20)	−1.35 to 3.34	0.407		
	Cesarean delivery	0.41 (1.73)	−2.98 to 3.80	0.813		
	EBF at survey	2.64 (1.68)	−0.64 to 5.93	0.116		
	EPDS	−0.23 (0.14)	−0.50 to 0.05	0.111		
	Social support	0.54 (0.12)	0.31 to 0.76	<0.001		
	Breastfeeding attitude	0.16 (0.08)	0.01 to 0.31	0.036		

*B* values are unstandardized coefficients. Reference categories were high school or below, primiparous, vaginal birth, and non-exclusive breastfeeding. BF, breastfeeding; EPDS, Edinburgh Postnatal Depression Scale.

SOC and BSES-SF remained positively associated with HHLS total score in the parsimonious model. In Model 4, SOC (*B* = 0.15, 95% CI 0.01 to 0.29), BSES-SF (*B* = 0.45, 95% CI 0.29 to 0.61), multiparity, social support, and IFAS were positively associated with HHLS total score. In standardized units, BSES-SF (*β* = 0.34) and social support (*β* = 0.28) had the largest coefficients. No established minimal clinically important difference is available for HHLS, so these estimates should not be translated directly into clinical benefit.

### 3.6 Indirect association through breastfeeding self-efficacy

Bootstrap path models identified a positive cross-sectional indirect association between SOC and HHLS total score through BSES-SF (Table 7). In the crude model, the indirect estimate was 0.203 (bootstrap 95% CI 0.110 to 0.315). In the parsimonious adjusted model, the total, direct, and indirect estimates were 0.430, 0.243, and 0.187, respectively; the indirect 95% CI was 0.097 to 0.299. These estimates describe a statistical decomposition and do not establish temporal ordering.

**Table 7:** Bootstrap cross-sectional path models for SOC, BSES-SF, and HHLS total score.

Model	Adjustment	Effect	Estimate	Bootstrap 95% CI	Descriptive ratio
Crude	No covariates	Total association (c)	0.441	0.270 to 0.615	
		Direct association (c′)	0.237	0.077 to 0.401	
		Indirect association (a × b)	0.203	0.110 to 0.315	46.2%
Core	Education, parity, expected breastfeeding duration	Total association (c)	0.430	0.261 to 0.602	
		Direct association (c′)	0.243	0.086 to 0.404	
		Indirect association (a × b)	0.187	0.097 to 0.299	43.5%
Expanded	Parsimonious + age, income, delivery, current feeding, EPDS, support, IFAS	Total association (c)	0.193	0.029 to 0.356	
		Direct association (c′)	0.149	−0.025 to 0.314	
		Indirect association (a × b)	0.044	−0.002 to 0.108	22.8%

Estimates are unstandardized cross-sectional associations based on 5,000 bootstrap resamples with a fixed random seed. Confidence limits are reported to three decimal places. “Core” denotes the parsimonious adjusted model. Descriptive ratios are not causal proportions.

The expanded model yielded a total association of 0.193, a direct association of 0.149, and an indirect estimate of 0.044 (bootstrap 95% CI -0.002 to 0.108). The interval therefore included zero when evaluated using the unrounded bootstrap limits. Figure 4 shows the parsimonious path model, and Figure 5 presents the corresponding bootstrap distribution and effect estimates. The standardized coefficients from the expanded regression model are shown in Figure 6.

**Figure 4:**
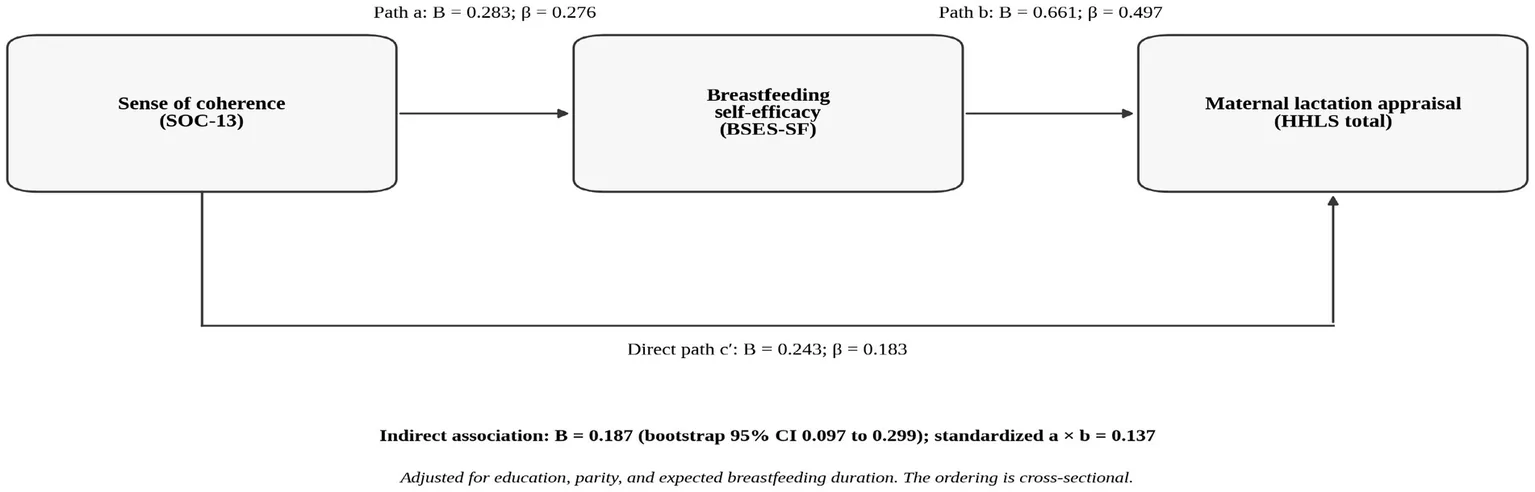
Parsimonious adjusted cross-sectional path model. Values are unstandardized coefficients (*B*) with standardized coefficients (*β*) shown in the figure. The model adjusted for education, parity, and expected breastfeeding duration; the ordering does not establish temporal or causal direction.

**Figure 5:**
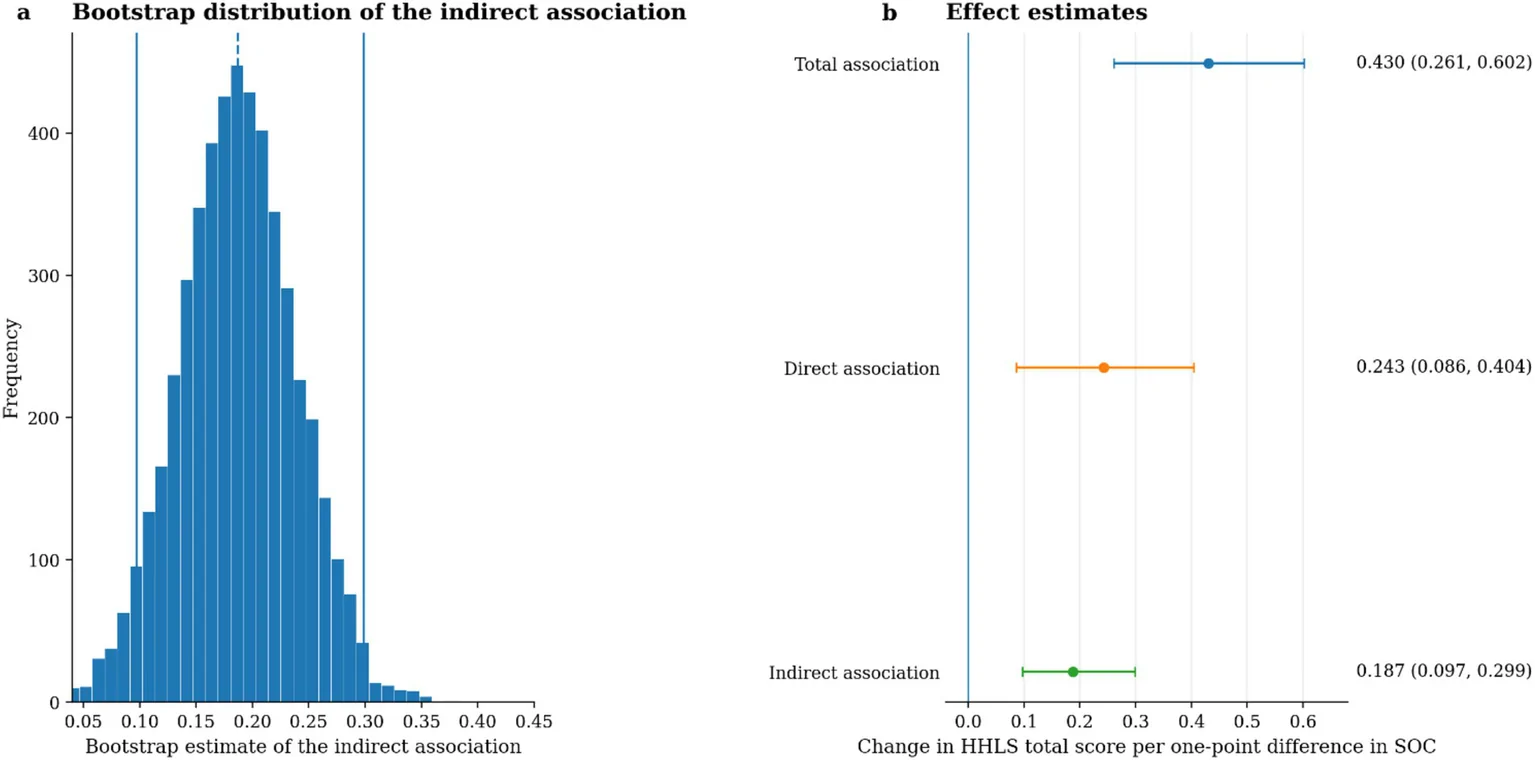
Bootstrap evidence for the parsimonious adjusted indirect association. Panel **(a)** shows the 5,000-resample bootstrap distribution and exact percentile limits. Panel **(b)** shows total, direct, and indirect estimates with 95% confidence intervals.

**Figure 6:**
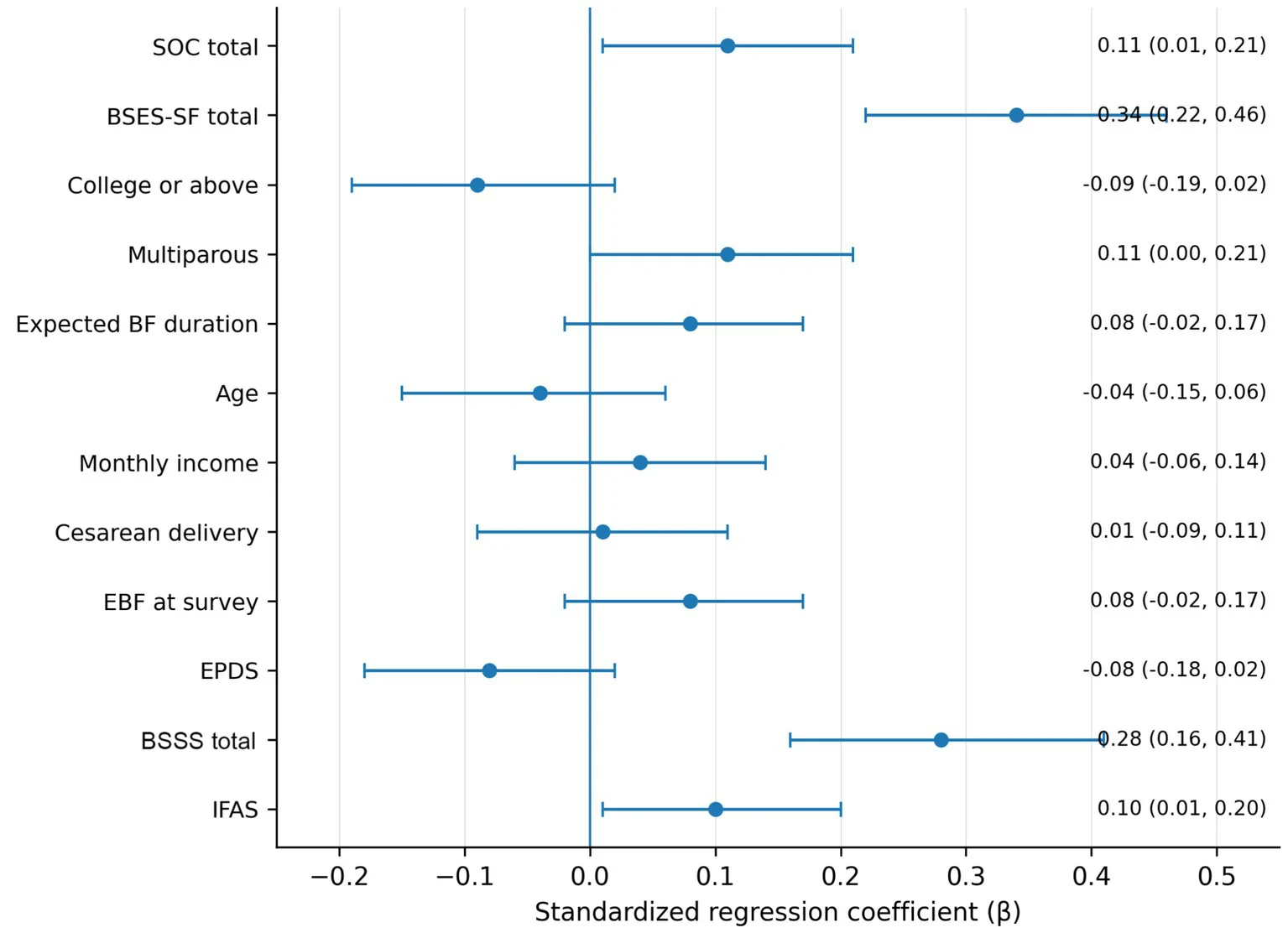
Standardized coefficients from the expanded regression model predicting HHLS total perception of milk supply. All model predictors are displayed; points show *β* coefficients and lines show 95% confidence intervals.

### 3.7 HHLS domain-specific path models

Domain-specific models clarified the content underlying the HHLS total-score result (Table 4). In the parsimonious model, the indirect association through BSES-SF was 0.086 (bootstrap 95% CI 0.036 to 0.152) for Maternal confidence/commitment and 0.102 (0.057 to 0.155) for Maternal-infant breastfeeding. For perceived infant satiety, the indirect estimate was −0.001 (−0.021 to 0.019).

The total-score association therefore reflected the confidence and satisfaction components of perception of milk supply rather than a clear association with perceived infant satiety. This does not invalidate the established multidimensional HHLS total score, but it narrows its interpretation and supports retaining “perception of milk supply” as the manuscript’s principal terminology.

### 3.8 Sensitivity analyses and model diagnostics

The result was not dependent on the inclusion of women currently formula feeding. After excluding these 48 participants, the indirect estimate was 0.199 (bootstrap 95% CI 0.102 to 0.323) in the parsimonious model and 0.061 (0.004 to 0.147) in the expanded model. A separate background-characteristic model that included age, ethnicity, education, pre-pregnancy BMI category, employment, residence, parity, and delivery mode yielded an indirect estimate of 0.192 (0.101 to 0.303). These sensitivity analyses are reported in Supplementary Table S3.

Model diagnostics did not indicate severe multicollinearity (VIF 1.04–1.69) or marked residual non-normality (Jarque-Bera *p* = 0.086). The Breusch-Pagan test indicated heteroscedasticity (*p* = 0.008); HC3-robust inference retained a clear BSES-SF coefficient in the expanded model (B = 0.452, 95% CI 0.271 to 0.632), whereas the SOC coefficient was less precise. Maximum Cook’s distance was 0.098; no observation approached 1. A sensitivity refit excluding the 18 observations above 4/n retained a positive BSES-SF coefficient (*B* = 0.526) but attenuated the SOC coefficient (*B* = 0.036).

Exploratory SOC dimension-specific indirect associations remained positive, but meaningfulness had low internal consistency (alpha = 0.592). These results are retained in Supplementary Table S2 for completeness and are not used to strengthen the primary total-score interpretation.

## 4 Discussion

### 4.1 Principal findings and construct interpretation

In this hospital-based sample, SOC, BSES-SF, and HHLS total scores were positively associated. BSES-SF added substantial explanatory value to the HHLS total-score model, and the parsimonious indirect estimate remained positive after women currently formula feeding were excluded. The domain models place a necessary boundary around this result: the association was evident for breastfeeding Maternal confidence/commitment and satisfaction, but not for perceived infant satiety. HHLS total is therefore most appropriately interpreted as a broad perception of milk supply rather than a direct measure of milk volume or infant intake.

This pattern is compatible with prior evidence linking breastfeeding self-efficacy to continuation, persistence, and satisfaction (73). It also shows why construct overlap must be considered rather than treated as a reason to dismiss the total score. BSES-SF and the HHLS confidence domain capture related but not identical judgments: one concerns perceived capability to breastfeed, while the other forms part of a broader appraisal that also includes satisfaction and infant cues. The domain models indicate that the observed indirect association concerns mothers’ confidence and evaluation of the lactation experience, not perceived satiety alone.

SOC remains relevant as a broad salutogenic orientation toward comprehensibility, manageability, and meaningfulness (74). Its association with breastfeeding confidence is consistent with research linking sense of coherence to perinatal adaptation (75). Social support was also strongly related to BSES-SF and HHLS total score, reinforcing an ecological interpretation in which confidence develops within family, clinical, cultural, and institutional contexts (76). These constructs should not be ranked as isolated competing explanations; they represent related levels of the postpartum feeding environment.

### 4.2 Model specification, concurrent covariates, and temporal ambiguity

The indirect estimate decreased from 0.187 in the parsimonious model to 0.044 in the expanded model, with the latter interval extending slightly below zero. This attenuation confirms that the estimate is sensitive to how concurrent mood, support, feeding attitudes, and current feeding status are handled. At the same time, a model restricted to background characteristics produced an estimate of 0.192, and the result remained positive after formula-feeding participants were excluded. The evidence therefore supports a reproducible cross-sectional association while remaining insufficient to identify a unique causal adjustment set.

Alternative temporal orderings remain plausible. Favorable breastfeeding experiences may strengthen BSES-SF through mastery experiences, while lower confidence may accompany supplementation or discontinuation. Maternal expectations, mood, support, and appraisal can also change reciprocally during the first postpartum weeks. Longitudinal measurement beginning before feeding outcomes are established is needed to separate these processes.

### 4.3 Feeding status, Qinghai context, and generalizability

Current feeding mode was recorded at the survey, but breastfeeding initiation, duration before cessation, reasons for discontinuation, and item applicability were not. This limits interpretation of responses from women currently formula feeding. Reassuringly, excluding those women did not remove the central indirect association and produced similar point estimates among the 207 women reporting exclusive or mixed feeding. These restricted-sample findings strengthen the internal consistency of the result without resolving whether self-efficacy preceded feeding experience.

The Qinghai setting also shapes interpretation. The hospital serves geographically dispersed and ethnically diverse communities; 39.2% of participants identified as ethnic minorities. Questionnaires were administered in Mandarin without separate minority-language versions, and culturally specific meanings of coping, family support, confidence, and infant satiety may not be fully equivalent across groups. The higher unadjusted HHLS total score among women with lower education should not be treated as protective. The subgroup was small, the adjusted coefficient was unclear, and parity, prior caregiving, family involvement, response style, or residual confounding may explain the pattern.

### 4.4 Public health and clinical implications

These results offer assessment context rather than evidence that BSES-SF can diagnose insufficient milk. BSES-SF may provide complementary information about maternal breastfeeding confidence when interpreted together with clinical lactation assessment. It should not substitute for evaluation of latch, milk transfer, maternal symptoms, infant growth, hydration, or other clinical indicators, and this study did not estimate screening accuracy or clinically useful thresholds.

From an international public health perspective, the findings support a broad view of perception of milk supply that includes confidence, satisfaction, infant cues, mood, and support. Service models that provide consistent information, include partners and family caregivers, and ensure timely clinical review are compatible with this interpretation. Whether confidence-oriented support changes infant satiety perceptions, supplementation, exclusivity, or duration must be tested prospectively rather than inferred from these cross-sectional associations.

### 4.5 Strengths and limitations

Strengths include use of established Chinese-language measures, direct reanalysis of participant-level data, exact bootstrap intervals, comparison of alternative adjustment sets, domain-specific path models, an active-breastfeeding sensitivity analysis, and formal model diagnostics. Several limitations remain. The cross-sectional design does not establish temporal ordering, and single-hospital recruitment limits external validity. Exact postpartum assessment days were not recorded. The participant log did not disaggregate the 25 questionnaires not included in analysis, and detailed breastfeeding histories were unavailable for formula-feeding participants.

All principal measures were self-reported at one assessment, creating potential common method variance, social desirability bias, and mood-congruent reporting. No minority-language versions were administered, so measurement equivalence across ethnic groups cannot be assumed despite use of Chinese-language instruments. HHLS was not validated against milk transfer, infant weight trajectory, or clinical lactation assessment, and its Maternal confidence/commitment domain overlaps conceptually with BSES-SF. The meaningfulness subscale of SOC had low internal consistency. Finally, heteroscedasticity and some influential observations were present, although robust and restricted-sample checks retained the central BSES-SF association.

## 5 Conclusion

In this single-center cross-sectional study, breastfeeding self-efficacy was consistently associated with mothers’ broader perception of milk supply, particularly breastfeeding Maternal confidence/commitment and satisfaction. The indirect association with SOC remained evident among women currently reporting exclusive or mixed feeding, but it was attenuated under expanded psychosocial adjustment and was not observed for perceived infant satiety. These findings support BSES-SF as complementary information about maternal confidence, not as a diagnostic measure of milk sufficiency. Longitudinal, multisite studies combining psychosocial measures with objective lactation indicators are needed.

## Data Availability

The datasets generated and analyzed during the current study are not publicly available because they contain participant-level information subject to ethical and privacy considerations. Requests to access the data may be directed to the corresponding author, Xuekun Zhang (zhangxuekun0115@suda.edu.cn), and will be considered in accordance with applicable ethical and institutional requirements.

## References

[1] World Health Organization Infant and young child Feeding Geneva World Health Organization (2023). Available online at: https://www.who.int/news-room/fact-sheets/detail/infant-and-young-child-feeding (Accessed August 26, 2026).

[2] UNICEF World Health Organization On World Breastfeeding Week, UNICEF and WHO call for equal Access to Breastfeeding Support Geneva World Health Organization (2024) Available online at: https://www.who.int/news/item/31-07-2024-on-world-breastfeeding-week--unicef-and-who-call-for-equal-access-to-breastfeeding-support (Accessed August 26, 2026).

[3] Victora CG Bahl R Barros AJD França GVA Horton S Krasevec J . Breastfeeding in the 21st century: epidemiology, mechanisms, and lifelong effect. Lancet. (2016) 387:475–90. doi: 10.1016/S0140-6736(15)01024-7, 26869575

[4] Rollins NC Bhandari N Hajeebhoy N Horton S Lutter CK Martines JC . Why invest, and what it will take to improve breastfeeding practices? Lancet. (2016) 387:491–504. doi: 10.1016/S0140-6736(15)01044-2, 26869576

[5] Perez-Escamilla R Tomori C Hernandez-Cordero S Baker P Barros AJD Begin F . Breastfeeding: crucially important, but increasingly challenged in a market-driven world. Lancet. (2023) 401:472–85. doi: 10.1016/S0140-6736(22)01932-8, 36764313

[6] Baker P Smith JP Garde A Grummer-Strawn LM Wood B Sen G . The political economy of infant and young child feeding: confronting corporate power, overcoming structural barriers, and accelerating progress. Lancet. (2023) 401:503–24. doi: 10.1016/S0140-6736(22)01933-X, 36764315

[7] North K Gao M Allen G Lee AC. Breastfeeding in a global context: epidemiology, impact, and future directions. Clin Ther. (2022) 44:228–44. doi: 10.1016/j.clinthera.2021.11.017, 34973827

[8] Meek JY Noble L. Policy statement: breastfeeding and the use of human milk. Pediatrics. (2022) 150:e2022057988. doi: 10.1542/peds.2022-057988, 35921640

[9] Kang L Liang J He C Miao L Li X Dai L . Breastfeeding practice in China from 2013 to 2018: a study from a national dynamic follow-up surveillance. BMC Public Health. (2021) 21:329. doi: 10.1186/s12889-021-10211-2, 33568130 PMC7874457

[10] National Health Commission of the People’s Republic of China. Action Plan to Improve Child Health, 2021–2025. Beijing: National Health Commission (2021).

[11] Gatti L. Maternal perceptions of insufficient milk supply in breastfeeding. J Nurs Scholarsh. (2008) 40:355–63. doi: 10.1111/j.1547-5069.2008.00234.x, 19094151 PMC4508856

[12] Huang Y Liu Y Yu XY Zeng TY. The rates and factors of perceived insufficient milk supply: a systematic review. Matern Child Nutr. (2022) 18:e13255. doi: 10.1111/mcn.13255, 34382733 PMC8710095

[13] Li R Fein SB Chen J Grummer-Strawn LM. Why mothers stop breastfeeding: mothers’ self-reported reasons for stopping during the first year. Pediatrics. (2008) 122:S69–76. doi: 10.1542/peds.2008-1315i, 18829834

[14] Odom EC Li R Scanlon KS Perrine CG Grummer-Strawn L. Reasons for earlier than desired cessation of breastfeeding. Pediatrics. (2013) 131:e726–32. doi: 10.1542/peds.2012-1295, 23420922 PMC4861949

[15] McCarter-Spaulding DE Kearney MH. Parenting self-efficacy and perception of insufficient breast milk. J Obstet Gynecol Neonatal Nurs. (2001) 30:515–22. doi: 10.1111/j.1552-6909.2001.tb01571.x, 11572532

[16] Otsuka K Dennis CL Tatsuoka H Jimba M. The relationship between breastfeeding self-efficacy and perceived insufficient milk among Japanese mothers. J Obstet Gynecol Neonatal Nurs. (2008) 37:546–55. doi: 10.1111/j.1552-6909.2008.00277.x, 18811774

[17] Sandhi A Lee GT Chipojola R Huda MH Kuo SY. The relationship between perceived milk supply and exclusive breastfeeding during the first six months postpartum: a cross-sectional study. Int Breastfeed J. (2020) 15:65. doi: 10.1186/s13006-020-00310-y, 32680551 PMC7367342

[18] De Roza JG Fong MK Ang BL Sadon RB Koh EYL Teo SSH. Exclusive breastfeeding, breastfeeding self-efficacy and perception of milk supply among mothers in Singapore: a longitudinal study. Midwifery. (2019) 79:102532. doi: 10.1016/j.midw.2019.102532, 31526969

[19] Galipeau R Dumas L Lepage M. Perception of not having enough milk and actual milk production of first-time breastfeeding mothers: is there a difference? Breastfeed Med. (2017) 12:210–7. doi: 10.1089/bfm.2016.0183, 28326807

[20] Antonovsky A. Health, Stress, and Coping. San Francisco, CA: Jossey-Bass (1979).

[21] Antonovsky A. Unraveling the Mystery of Health: How People Manage Stress and Stay Well. San Francisco, CA: Jossey-Bass (1987).

[22] Eriksson M Lindström B. Validity of Antonovsky’s sense of coherence scale: a systematic review. J Epidemiol Community Health. (2005) 59:460–6. doi: 10.1136/jech.2003.018085, 15911640 PMC1757043

[23] Eriksson M Lindström B. Antonovsky’s sense of coherence scale and the relation with health: a systematic review. J Epidemiol Community Health. (2006) 60:376–81. doi: 10.1136/jech.2005.041616, 16614325 PMC2563977

[24] Super S Wagemakers MAE Picavet HSJ Verkooijen KT Koelen MA. Strengthening sense of coherence: opportunities for theory building in health promotion. Health Promot Int. (2016) 31:869–78. doi: 10.1093/heapro/dav071, 26208654

[25] Sierra-Garcia E Saus-Ortega C. Sense of coherence in breastfeeding women: a scoping review. An Sist Sanit Navar. (2024) 47:e1064. doi: 10.23938/ASSN.1064, 38349139 PMC10913710

[26] Cortelo FM Marba STM Cortellazzi KL Ambrosano GMB Guerra LM Almeida AC . Women’s sense of coherence and its association with early weaning. J Pediatr. (2018) 94:624–9. doi: 10.1016/j.jped.2017.08.007 29097193

[27] Thomson G Dykes F. Women’s sense of coherence related to their infant feeding experiences. Matern Child Nutr. (2011) 7:160–74. doi: 10.1111/j.1740-8709.2010.00251.x, 21108741 PMC6860619

[28] Voogand M Alehagen S Salomonsson B. The relationship between fear of childbirth and sense of coherence in women expecting their first child. Sex Reprod Healthc. (2020) 26:100555. doi: 10.1016/j.srhc.2020.100555, 33130537

[29] Bandura A. Self-efficacy: toward a unifying theory of behavioral change. Psychol Rev. (1977) 84:191–215. doi: 10.1037/0033-295X.84.2.191 847061

[30] Bandura A. Self-Efficacy: The Exercise of Control. New York, NY: W.H. Freeman (1997).

[31] Dennis CL Faux S. Development and psychometric testing of the breastfeeding self-efficacy scale. Res Nurs Health. (1999) 22:399–409. doi: 10.1002/(SICI)1098-240X(199910)22:5<399::AID-NUR6>3.0.CO;2-4, 10520192

[32] Dennis CL. The breastfeeding self-efficacy scale: psychometric assessment of the short form. J Obstet Gynecol Neonatal Nurs. (2003) 32:734–44. doi: 10.1177/0884217503258459 14649593

[33] Dennis CL McQueen K Dol J Brown H Beck CT Shorey S. Psychometrics of the breastfeeding self-efficacy scale and short form: a systematic review. BMC Public Health. (2024) 24:637. doi: 10.1186/s12889-024-17805-6, 38419045 PMC10903029

[34] Brockway M Benzies K Hayden KA. Interventions to improve breastfeeding self-efficacy and resultant breastfeeding rates: a systematic review and meta-analysis. J Hum Lact. (2017) 33:486–99. doi: 10.1177/0890334417707957 28644764

[35] Chipojola R Chiu HY Huda MH Lin YM Kuo SY. Effectiveness of theory-based educational interventions on breastfeeding self-efficacy and exclusive breastfeeding: a systematic review and meta-analysis. Int J Nurs Stud. (2020) 109:103675. doi: 10.1016/j.ijnurstu.2020.103675, 32585447

[36] Sinha B Chowdhury R Sankar MJ Martines J Taneja S Mazumder S . Interventions to improve breastfeeding outcomes: a systematic review and meta-analysis. Acta Paediatr. (2015) 104:114–34. doi: 10.1111/apa.13127 26183031

[37] McFadden A Gavine A Renfrew MJ Wade A Buchanan P Taylor JL . Support for healthy breastfeeding mothers with healthy term babies. Cochrane Database Syst Rev. (2017) 2017:CD001141. doi: 10.1002/14651858.CD001141.pub5, 28244064 PMC6464485

[38] Demirci JR Suffoletto B Doman J Glasser M Chang JC Sereika SM . The development and evaluation of a text message program to prevent perceived insufficient milk among first-time mothers: retrospective analysis of a randomized controlled trial. JMIR Mhealth Uhealth. (2020) 8:e17328. doi: 10.2196/17328, 32347815 PMC7221632

[39] Hill PD Humenick SS. Development of the H&H lactation scale. Nurs Res. (1996) 45:136–40. doi: 10.1097/00006199-199605000-00003 8637793

[40] Huang YY Lee JT Huang CM Gau ML. Factors related to maternal perception of milk supply while in the hospital. J Nurs Res. (2009) 17:179–88. doi: 10.1097/JNR.0b013e3181b25558, 19738446

[41] Dias CC Figueiredo B. Breastfeeding and depression: a systematic review of the literature. J Affect Disord. (2015) 171:142–54. doi: 10.1016/j.jad.2014.09.022 25305429

[42] Scott JA Shaker I Reid M. Parental attitudes toward breastfeeding: their association with feeding outcome at hospital discharge. Birth. (2004) 31:125–31. doi: 10.1111/j.0730-7659.2004.00290.x, 15153132

[43] Meedya S Fahy K Kable A. Factors that positively influence breastfeeding duration to 6 months: a literature review. Women Birth. (2010) 23:135–45. doi: 10.1016/j.wombi.2010.02.002, 20299299

[44] La Rosa VL Commodari E. Breastfeeding self-efficacy in Italian mothers: the role of attachment, parental stress, and feeding practices. BMC Psychol. (2026) 14:822. doi: 10.1186/s40359-026-04586-3, 42026677 PMC13237887

[45] von Elm E Altman DG Egger M Pocock SJ Gøtzsche PC Vandenbroucke JP. The Strengthening the Reporting of Observational Studies in Epidemiology (STROBE) Statement: Guidelines for Reporting Observational Studies. PLoS Med. (2007) 4:e296. doi: 10.1371/journal.pmed.0040296 PMC2020495 17941714

[46] Ding Y Bao LP Xu H Huo Y Rahm Hallberg I. Psychometric properties of the Chinese version of sense of coherence scale in women with cervical cancer. Psychooncology. (2012) 21:1205–14. doi: 10.1002/pon.2029 21823198

[47] Eriksson M Mittelmark MB. "The sense of coherence and its measurement". In: Mittelmark MB Sagy S Eriksson M Bauer GF Pelikan JM Lindström B , editors. The Handbook of Salutogenesis. Cham: Springer (2017). p. 97–106.

[48] Ip WY Gao LL Choi KC Chau JPC Xiao Y. The short form of the breastfeeding self-efficacy scale as a prognostic factor of exclusive breastfeeding among mandarin-speaking Chinese mothers. J Hum Lact. (2016) 32:711–20. doi: 10.1177/0890334416658014, 27474407

[49] Strecher VJ DeVellis BM Becker MH Rosenstock IM. The role of self-efficacy in achieving health behavior change. Health Educ Q. (1986) 13:73–92. doi: 10.1177/109019818601300108, 3957687

[50] Punthmatharith B Singh J. A psychometric assessment of the H&H Lactation Scale in a sample of Thai mothers using a repeated measurement design. Nurs Res. (2005) 54:313–23. doi: 10.1097/00006199-200509000-00005, 16224317

[51] Zhang F Yang Y Bai T Sun L Sun M Shi X . Effect of pumping pressure on onset of lactation after caesarean section: a randomized controlled study. Matern Child Nutr. (2018) 14:e12486. doi: 10.1111/mcn.12486, 28752645 PMC6866149

[52] Lau Y Wang Y Yin L Chan KS Guo X. Validation of the mainland Chinese version of the Edinburgh postnatal depression scale in Chengdu mothers. Int J Nurs Stud. (2010) 47:1139–51. doi: 10.1016/j.ijnurstu.2010.02.005, 20219196

[53] Dai HX Guan XD Li XM You LM Lau Y. Psychometric properties of a mainland Chinese version of the Iowa infant feeding attitude scale among postpartum women in China. Contemp Nurse. (2013) 44:11–20. doi: 10.5172/conu.2013.44.1.11, 23721383

[54] Sun T. Chinese adaptation and application of the Breastfeeding Social Support Scale [master’s thesis]. Chengde Medical University. (2024).

[55] Tuthill EL McGrath JM Graber M Cusson RM Young SL. Breastfeeding self-efficacy: a critical review of available instruments. J Hum Lact. (2016) 32:35–45. doi: 10.1177/0890334415599533, 26319113 PMC4882127

[56] Balogun OO Dagvadorj A Anigo KM Ota E Sasaki S. Factors influencing breastfeeding exclusivity during the first 6 months of life in developing countries: a quantitative and qualitative systematic review. Matern Child Nutr. (2015) 11:433–51. doi: 10.1111/mcn.12180, 25857205 PMC6860250

[57] McKinney CO Hahn-Holbrook J Chase-Lansdale PL Ramey SL Krohn J Reed-Vance M . Racial and ethnic differences in breastfeeding. Pediatrics. (2016) 138:e20152388. doi: 10.1542/peds.2015-2388, 27405771 PMC4960721

[58] Valero-Chillerón MJ Mena-Tudela D Cervera-Gasch Á González-Chordá VM Soriano-Vidal FJ Quesada JA . Influence of health literacy on maintenance of exclusive breastfeeding at 6 months postpartum: a multicentre study. Int J Environ Res Public Health. (2022) 19:5411. doi: 10.3390/ijerph19095411, 35564807 PMC9104596

[59] Dagher RK McGovern PM Schold JD Randall XJ. Determinants of breastfeeding initiation and cessation among employed mothers: a prospective cohort study. BMC Pregnancy Childbirth. (2016) 16:194. doi: 10.1186/s12884-016-0965-1, 27472915 PMC4966748

[60] Turcksin R Bel S Galjaard S Devlieger R. Maternal obesity and breastfeeding intention, initiation, intensity and duration: a systematic review. Matern Child Nutr. (2014) 10:166–83. doi: 10.1111/j.1740-8709.2012.00439.x, 22905677 PMC6860286

[61] Ngo LTH Chou HF Gau ML Liu CY. Breastfeeding self-efficacy and related factors in postpartum Vietnamese women. Midwifery. (2019) 70:84–91. doi: 10.1016/j.midw.2018.12.014, 30594613

[62] Lau CYK Lok KYW Tarrant M. Breastfeeding duration and the theory of planned behavior and breastfeeding self-efficacy framework: a systematic review of observational studies. Matern Child Health J. (2018) 22:327–42. doi: 10.1007/s10995-018-2453-x, 29427014

[63] Prior E Santhakumaran S Gale C Philipps LH Modi N Hyde MJ. Breastfeeding after cesarean delivery: a systematic review and meta-analysis of world literature. Am J Clin Nutr. (2012) 95:1113–35. doi: 10.3945/ajcn.111.030254, 22456657

[64] Awaliyah SN Rachmawati IN Rahmah H. Breastfeeding self-efficacy as a dominant factor affecting maternal breastfeeding satisfaction. BMC Nurs. (2019) 18:30. doi: 10.1186/s12912-019-0359-6, 31427892 PMC6696667

[65] Ystrom E. Breastfeeding cessation and symptoms of anxiety and depression: a longitudinal cohort study. BMC Pregnancy Childbirth. (2012) 12:36. doi: 10.1186/1471-2393-12-36, 22621668 PMC3449190

[66] DiGirolamo A Thompson N Martorell R Fein S Grummer-Strawn L. Intention or experience? Predictors of continued breastfeeding. Health Educ Behav. (2005) 32:208–26. doi: 10.1177/1090198104271971, 15749967

[67] Maleki-Saghooni N Amel Barez M Karimi FZ. Investigation of the relationship between social support and breastfeeding self-efficacy in primiparous breastfeeding mothers. J Matern Fetal Neonatal Med. (2020) 33:3097–102. doi: 10.1080/14767058.2019.1568986, 30632820

[68] Schisterman EF Cole SR Platt RW. Overadjustment bias and unnecessary adjustment in epidemiologic studies. Epidemiology. (2009) 20:488–95. doi: 10.1097/EDE.0b013e3181a819a1, 19525685 PMC2744485

[69] VanderWeele TJ. Mediation analysis: a practitioner’s guide. Annu Rev Public Health. (2016) 37:17–32. doi: 10.1146/annurev-publhealth-032315-021402, 26653405

[70] Norman G. Likert scales, levels of measurement and the “laws” of statistics. Adv Health Sci Educ Theory Pract. (2010) 15:625–32. doi: 10.1007/s10459-010-9222-y, 20146096

[71] Sterne JAC White IR Carlin JB Spratt M Royston P Kenward MG . Multiple imputation for missing data in epidemiological and clinical research: potential and pitfalls. BMJ. (2009) 338:b2393. doi: 10.1136/bmj.b2393, 19564179 PMC2714692

[72] White IR Royston P Wood AM. Multiple imputation using chained equations: issues and guidance for practice. Stat Med. (2011) 30:377–99. doi: 10.1002/sim.4067, 21225900

[73] Blyth R Creedy DK Dennis CL Moyle W Pratt J De Vries SM. Effect of maternal confidence on breastfeeding duration: an application of breastfeeding self-efficacy theory. Birth. (2002) 29:278–84. doi: 10.1046/j.1523-536X.2002.00202.x, 12484390

[74] Lindström B Eriksson M. Salutogenesis. J Epidemiol Community Health. (2005) 59:440–2. doi: 10.1136/jech.2005.034777, 15911636 PMC1757059

[75] Ferguson SL Davis D Browne J Taylor J. Sense of coherence and childbearing: a scoping review of the literature. Int J Childbirth. (2014) 4:134–50. doi: 10.1891/2156-5287.4.3.134

[76] de Jager E Skouteris H Broadbent J Amir LH Mellor K. Psychosocial correlates of exclusive breastfeeding: a systematic review. Midwifery. (2013) 29:506–18. doi: 10.1016/j.midw.2012.04.009, 23099152

