# Supplementary material for: Breastfeeding self-efficacy as a mediator between sense of coherence and perception of milk supply among postpartum women: a cross-sectional study
Source: Front Public Health. 2026 Sep 18;14:1894005. doi: 10.3389/fpubh.2026.1894005 (PMC13630934; doi:10.3389/fpubh.2026.1894005)
Supplement: Supplementary file 1 [file Supplementary_file_1.docx]

Supplementary Material

**Supplementary Table S1. Expanded domain-level correlations with BSES-SF and HHLS outcomes.**

| **Predictor** | **BSES total** | **HHLS total** | **HHLS maternal confidence/commitment** | **HHLS perceived infant satiety** | **HHLS maternal-infant breastfeeding satisfaction** |
| --- | --- | --- | --- | --- | --- |
| SOC comprehensibility | 0.26 (<0.001) | 0.28 (<0.001) | 0.26 (<0.001) | 0.11 (0.073) | 0.24 (<0.001) |
| SOC manageability | 0.24 (<0.001) | 0.30 (<0.001) | 0.29 (<0.001) | 0.10 (0.116) | 0.25 (<0.001) |
| SOC meaningfulness | 0.27 (<0.001) | 0.30 (<0.001) | 0.32 (<0.001) | 0.07 (0.264) | 0.24 (<0.001) |
| BSES technical | 0.97 (<0.001) | 0.58 (<0.001) | 0.47 (<0.001) | 0.06 (0.354) | 0.76 (<0.001) |
| BSES intrapersonal | 0.97 (<0.001) | 0.54 (<0.001) | 0.46 (<0.001) | 0.04 (0.564) | 0.72 (<0.001) |
| Breastfeeding social support (BSSS) | 0.60 (<0.001) | 0.54 (<0.001) | 0.52 (<0.001) | 0.00 (0.998) | 0.63 (<0.001) |
| Breastfeeding attitude | 0.08 (0.189) | 0.16 (0.011) | 0.16 (0.012) | 0.08 (0.217) | 0.11 (0.089) |
| EPDS depressive symptoms | 0.07 (0.287) | -0.07 (0.298) | -0.04 (0.577) | -0.20 (0.001) | 0.08 (0.217) |

*Note: Values are Pearson correlation coefficients, with p values in parentheses. These analyses are exploratory. BSSS, Breastfeeding Social Support Scale. HHLS total represents a broad maternal perception of lactation.*

**Supplementary Table S2. Standardized regression coefficients, VIFs, and exploratory SOC dimension-specific indirect associations.**

| **Analysis** | **Term or pathway** | **Estimate (SE)** | **95% CI** | **p/value** | **Diagnostic or descriptive ratio** |
| --- | --- | --- | --- | --- | --- |
| Standardized regression | SOC total | 0.11 (0.05) | 0.01 to 0.21 | 0.040 | VIF 1.23 |
| Standardized regression | BSES-SF total | 0.34 (0.06) | 0.22 to 0.46 | <0.001 | VIF 1.69 |
| Standardized regression | College or above | -0.09 (0.05) | -0.19 to 0.02 | 0.099 | VIF 1.17 |
| Standardized regression | Multiparous | 0.11 (0.05) | 0.00 to 0.21 | 0.044 | VIF 1.20 |
| Standardized regression | Expected BF duration | 0.08 (0.05) | -0.02 to 0.17 | 0.109 | VIF 1.04 |
| Standardized regression | Age | -0.04 (0.05) | -0.15 to 0.06 | 0.408 | VIF 1.26 |
| Standardized regression | Monthly income | 0.04 (0.05) | -0.06 to 0.14 | 0.407 | VIF 1.08 |
| Standardized regression | Cesarean delivery | 0.01 (0.05) | -0.09 to 0.11 | 0.813 | VIF 1.09 |
| Standardized regression | EBF at survey | 0.08 (0.05) | -0.02 to 0.17 | 0.116 | VIF 1.04 |
| Standardized regression | EPDS | -0.08 (0.05) | -0.18 to 0.02 | 0.111 | VIF 1.10 |
| Standardized regression | BSSS total | 0.28 (0.06) | 0.16 to 0.41 | <0.001 | VIF 1.69 |
| Standardized regression | Breastfeeding attitude | 0.10 (0.05) | 0.01 to 0.20 | 0.036 | VIF 1.05 |
| Exploratory dimension-specific indirect association | Comprehensibility → BSES → HHLS | 0.43 | 0.23 to 0.66 | Bootstrap | Descriptive indirect-to-total association ratio: 47.0% |
| Exploratory dimension-specific indirect association | Manageability → BSES → HHLS | 0.43 | 0.17 to 0.71 | Bootstrap | Descriptive indirect-to-total association ratio: 40.5% |
| Exploratory dimension-specific indirect association | Meaningfulness → BSES → HHLS | 0.59 | 0.29 to 0.96 | Bootstrap | Descriptive indirect-to-total association ratio: 45.6% |

Note: Standardized regression estimates are reported with standard errors. VIF, variance inflation factor. EBF, exclusive breastfeeding; BSSS, Breastfeeding Social Support Scale. The SOC dimension-specific indirect associations are exploratory; the reported indirect-to-total association ratios are descriptive statistics and are not causal mediated proportions. The meaningfulness estimate requires particular caution because internal consistency was low.

**Supplementary Table S3. Sensitivity analyses for background adjustment and current feeding status.**

| Sensitivity analysis | n | Indirect association | Bootstrap 95% CI |
| --- | --- | --- | --- |
| Background-characteristic adjusted model | 255 | 0.192 | 0.101 to 0.303 |
| Active breastfeeding: parsimonious adjusted | 207 | 0.199 | 0.102 to 0.323 |
| Active breastfeeding: expanded adjusted | 207 | 0.061 | 0.004 to 0.147 |

Note: All entries are unstandardized indirect associations estimated with 5,000 bootstrap resamples. The active-breastfeeding sample excludes women reporting current formula feeding.

**Supplementary Table S4. Coding of predictors in the regression models.**

| Variable | Analysis form | Coding | Analytic role |
| --- | --- | --- | --- |
| SOC-13 total | Continuous | 13-91; one-point increments | Primary exposure |
| BSES-SF total | Continuous | 14-70; one-point increments | Statistical intermediary |
| Education | Binary | 0 = high school or below; 1 = college or above | Background characteristic |
| Parity | Binary | 0 = primiparous; 1 = multiparous | Background/reproductive history |
| Expected breastfeeding duration | Ordinal score | 1 = <6 months; 2 = 6-12 months; 3 = 1-2 years; 4 = ≥2 years | Concurrent intention; equal-spacing assumption |
| Age | Continuous | Years | Background characteristic |
| Monthly household income | Ordinal score | 1 = ≤5,000 CNY; 2 = 5,001-9,999 CNY; 3 = ≥10,000 CNY | Background socioeconomic measure; equal-spacing assumption |
| Mode of delivery | Binary | 0 = vaginal birth; 1 = cesarean birth | Obstetric characteristic |
| EBF at survey | Binary in expanded model; three-category descriptive variable | Regression: 0 = mixed or formula feeding; 1 = exclusive breastfeeding. Sensitivity: formula feeding excluded. | Current feeding correlate/downstream variable |
| EPDS total | Continuous | 0-30 | Concurrent psychosocial variable |
| BSSS total | Continuous | 11-55 | Concurrent psychosocial variable |
| IFAS total | Continuous | 17-85 | Concurrent psychosocial variable |

**Supplementary Figure S1. Participant flow during recruitment from April to July 2025.**

Note: The study log recorded 280 questionnaires distributed and 255 complete questionnaires included in analysis. It did not distinguish non-return from incomplete or inconsistent questionnaires among the 25 not included; these records are therefore reported collectively.
